# Topical vancomycin reduces the cerebrospinal fluid shunt infection rate: A retrospective cohort study

**DOI:** 10.1371/journal.pone.0190249

**Published:** 2018-01-09

**Authors:** Erik J. van Lindert, Martine van Bilsen, Michiel van der Flier, Eva Kolwijck, Hans Delye, Jaap ten Oever

**Affiliations:** 1 Department of Neurosurgery, Radboud University Medical Center, Nijmegen, The Netherlands; 2 Department of Pediatrics, Radboud University Medical Center, Nijmegen, The Netherlands; 3 Department of Medical Microbiology, Radboud University Medical Center, Nijmegen, The Netherlands; 4 Department of Internal Medicine, and Radboud Center for Infectious Diseases, Radboud University Medical Center, Nijmegen, The Netherlands; University of Toronto, CANADA

## Abstract

**Object:**

Despite many efforts at reduction, cerebrospinal fluid (CSF) shunt infections are a major cause of morbidity in shunt surgery, occurring in 5–15% of cases. To attempt to reduce the shunt infection rate at our institution, we added topical vancomycin (intrashunt and perishunt) to our existing shunt infection prevention protocol in 2012.

**Methods:**

We performed a retrospective cohort study comparing all shunted patients in January 2010 to December 2011 without vancomycin (control group, 263 procedures) to all patients who underwent shunt surgery between April 2012 and December 2015 with vancomycin (intervention group, 499 procedures).

**Results:**

The overall shunt infection rate significantly decreased from 6.8% (control group) to 3.0% (intervention group) (p = 0.023, absolute risk reduction 3.8%, relative risk reduction 56%). Multivariate logistic regression analysis confirmed that the addition of topical vancomycin showed that cases treated under a protocol of topical vancomycin were associated with a decreased shunt infection rate (odds ratio [OR] 0.49 95% CI 0.25–0.998; p = 0.049). Age < 1 year was associated with an increased risk of infection (OR) 4.41, 95% CI 2,10–9,26; p = 0.001). Time from surgery to infection was significantly prolonged in the intervention group (p = 0.001).

**Conclusion:**

Adding intraoperative vancomycin to a shunt infection prevention protocol significantly reduces CSF shunt infection rate.

## Introduction

The treatment of hydrocephalus consists of inserting a cerebrospinal fluid (CSF) shunt to the peritoneal or pleural cavities or to the atrium of the heart in the majority of cases. This insertion of foreign body materials may be accompanied by many potential problems, of which shunt infection is a major one. The average shunt shunt infection rate is about 5–15%, although lower and higher rates are reported [1–8]. Shunt infections are largely due to skin flora colonization of the shunt device at surgery, as 90% of infections are caused by *Staphylococcus* species, and most infections occur within the first few months after surgery [9]. The impact of a shunt infection is very high. An infection usually leads to shunt explantation and subsequent re-insertion of a new shunt, leading to prolonged hospitalization of weeks, which is accompanied by very high extra costs, and most importantly, it may have deleterious neurological effects.

Many efforts have been taken to reduce the shunt infection rate. Preoperative intravenous (iv) prophylactic antibiotics are administered routinely and this is a mainstay of prevention [10]. Many different measures appear to contribute to reducing the shunt infection rate, although evidence for each separate measure is minimal or nonexistent [11]. Combining of all these measures into an institutional shunt infection prevention protocol is the most effective means of preventing shunt infections [5]. The latest development is the use of antibiotic-impregnated catheters (AIC), with the prolonged release of two different antibiotics. Several meta-analyses have shown that AIC significantly reduce the shunt infection rate [6, 9, 12–14]. The use of AIC comes with significant extra costs and there are some concerns that it may increase the development of antibiotic resistance.

After its introduction in 2004, the use of a shunt infection protocol reduced the shunt infection rate in our department from 12% to 8% in our pediatric patient cohort. To further decrease the shunt infection rate and motivated by the results of Ragel et al., who used intrathecal antibiotics during surgery [15], we added a single new component to our existing protocol. A vancomycin solution of 2 mg/ml was used to drench, flush, and fill catheters, reservoirs, and valves during shunt surgery from April 2012 onwards. This study was performed to evaluate the effect of the addition of vancomycin to the existing protocol on the shunt infection rate. In addition we assesses (differences in) the time to infection and etiology.

## Methods

The work described has been carried out in accordance with The Code of Ethics of the World Medical Association (Declaration of Helsinki) for experiments involving humans. The study has been approved by the institutional review board CMO Arnhem—Nijmegen (IRB). The IRB waived informed consent due to the nature of the investigations.

This was a retrospective cohort study of all consecutive patients receiving any type of CSF shunt, first implantation, or shunt revision, from January 1, 2010, until 31 December 31, 2011, and from April 1, 2012, until December 31, 2015. This included patients with previous shunt infections and complicated shunt surgery. CSF reservoirs, stents, and external CSF drains were excluded. All patients underwent surgery at the Radboud University Medical Center department of neurosurgery.

Two cohorts containing consecutive cases were selected. The first (control group) was a consecutive series of all patients treated in 2010 and 2011 according to the institutional shunt infection prevention protocol introduced in 2004 and updated in 2008. In January 2012, vancomycin was introduced as an add-on to the existing protocol in a test phase for patients treated only by the first author (EJvL). From April 1, 2012, onwards, vancomycin was included as part of the new protocol for all patients. All patients receiving a shunt or a shunt revision between January 1 and 31 March 31, 2012, i.e., during the try-out phase, were therefore excluded. The second cohort (intervention group) therefore contained all patients treated between April 1, 2012, and December 31, 2015, and who received vancomycin in the new protocol.

Although the protocol is very strict, protocol violations could not be excluded, as protocol compliance checks were not performed.

A shunt was considered infected if the patient showed clinical signs of wound infection, septicemia in patients with ventriculoatrial (VA) shunts, peritonitis in patients with ventriculoperitoneal (VP) shunts, or meningitis, and if bacteria were cultivated from the blood, peritoneum, CSF, or the shunt system. A negative culture with typical positive clinical signs and CSF pleocytosis suggesting infection was also considered a shunt infection. Only shunt infections within 6 months after surgery (compliant with most of the literature) were collected; infections < 30 days after surgery were considered early infections.

### Shunt infection protocol and vancomycin

Our department has used a strict CSF shunt protocol since 2004, which was updated in 2008. Table 1 summarizes this protocol. Although the measures are strict, we do not have a protocol compliance checklist. Protocol violations did occur initially, but updates to the protocol and crew resource management training have minimized this.

**Table 1:** Radboud University Medical Center shunt infection prevention protocol, version 2012.

**OR planning**	1. Neonates/infants before older patients
	2. Single-bed patient rooms
**OR room**	1. Minimize number of staff in OR
	2. All potential material/implants in OR
	3. Closed doors from incision until wound closure
	4. OR door sign: “Keep out–shunt surgery”
**Patient preparation**	1. Iodine shampoo twice before surgery
	2. Regular pillow cleaning before and after surgery
	3. Cefazolin 30 minutes before surgery: children 25mg/kg, adults 2g
	4. Haircutting only with electric razor; in babies with shaving razor
	5. Skin disinfection by surgeon
	6. Use of iodine-impregnated skin drape (unless allergic to iodine)
	7. Shield from anesthesiological setup by drapes
	8. Maintain continuity between sterile draping and surgical instrumentation tables
	9. Create a gutter of drapes around patient, preventing instruments sliding from patient
	10. Bowl containing 250 ml 0.9% NaCl + 500 mg vancomycin on table
	11. Immerse implants and gauze before use in bowl
**Operative procedure**	1. Experienced neurosurgeon or resident under strict supervision
	2. Keep surgical time as short as possible
	3. Double gloving from the start
	4. First perform all skin incisions, dissection, and burr hole before unpacking implants, minimizing air exposure of implants
	5. Remove outer gloves before unpacking implants
	6. Unpacked implants immersed directly in vancomycin solution, assembled if necessary, and immediately implanted into the patient
	7. Flush a new shunt piece, do not test extensively
	8. Touch implants as little as possible with gloves, rather use instruments
	9. Cover open wounds with drenched gauze (vancomycin solution) as much as possible
	10. Careful hemostasis
	11. Careful wound closure, prevent necrosis
**Postoperative**	1. Adequate positioning, avoid local wound pressure (especially in babies and infants)
	2. Do not change bandages first 24 hours after surgery; shower after 72 hours

The use of intraoperative vancomycin was added to the above infection prevention measures in April 2012. Vancomycin was selected because intraoperative contamination by skin bacteria is the main route of transmission in shunt infection. Vancomycin (500 mg) is added to 250 ml 0.9% NaCl to create a solution of 2 mg/ml vancomycin. The solution is used to wet operative gauze, drench and flush the shunt (outside of the patient) and fill the shunt reservoir, flush the surgical wound superficially, and to wet surgical gloves. Vancomycin is normally not flushed into the ventricles or spinal thecal sac. The chosen concentration of vancomycin prevents overdosing in even the youngest children.

### Data collection and statistical analysis

The data of children aged <17 years were retrieved from a prospective database of complication registrations in pediatric neurosurgery that was started in our department in 2004. The data on patients aged >16 years were retrospectively collected from a digital surgical procedure registration system and digital patient files. All data were collected in an Excel database.

Statistical analysis was carried out using two-sided Fisher’s exact test and chi-square test for categorical outcomes. The primary outcome, differences in shunt infection rate before and after the addition of topical vancomycin, was assessed with logistic regression analysis with infection within 6 months as dependent variable. Patient factors showing an association with infection in univariate analysis at a significance level of p<0.1 were included in the multivariate model.

To study the effect of addition of vancomycin to the protocol on the time of infection, we carried out a multivariable cox proportional hazards regression to compare the time to infection using the same factors as in the multivariate logistic regression analysis.

Analysis was performed using commercially available statistical software (IBM SPSS Statistics for Windows, Version 22.0, IBM Corp: Armonk, NY). A p-value less than 0.05 was considered statistically significant.

## Results

### Patient population

In the first cohort of patients from 2010–2011 (control group), we studied 263 procedures, of which 131 were performed in children (<17 years) (Table 2). In children, the male-to-female (M/F) ratio was 1.08; that of adults was 0.65. The average age of pediatric patients was 6.1 (± 5.5) years; that of adults was 45 (± 20) years. The ratio of first shunt versus shunt revision was 0.54 and 1.13 in children and in adults, respectively, which reflects a much higher revision rate in children versus adults. A total of 37 procedures were performed in premature neonates (gestational age <36 weeks), 21 of which in the intervention group. Of the 263 procedures in the control group, 116 were new shunts and 147 shunt revisions. This was in the intervention group 222 and 277, respectively. Of all procedures 246 were performed by residents and 516 by neurosurgical staff members with an equal distribution in both study cohorts.

**Table 2:** Patient population and infection rates.

		Procedures		Infections		P-value
		No.	%	No.	%	
Total		762		33	4.3	
Gender	male	356	46.7	20	5.6	
	female	406	53.3	13	3.2	ns
Age	<1 year	99	13.0	13	13.1	0.000
	1–17 years	230	30.2	7	3.0	
	>17 years	433	56.8	13	3.0	
Prematurity	yes	37	37.4	6	16.2	
	no	62	62.6	7	11.3	ns
Protocol	control group	263	34.5	18	6.8	
	Intervention group	499	65.5	15	3.0	0.023
Surgeon	staff	516	67.7	25	4.8	
	resident	246	32.3	8	3.3	ns
Previous surgery <30 days	yes	132	17.3	10	7.6	
	no	630	82.7	23	3.7	0.057
Type of surgery	new shunt	338	44.4	11	3.3	
	revision	424	55.6	22	5.2	ns
Etiology	aqueductal stenosis	15	2.0	0	0	
	congenital	92	12.1	5	5.4	
	dysrafia	113	14.8	6	5.3	
	IIH	74	9.7	0	0	
	NPH	72	9.4	0	0	
	miscellaneous	43	5.6	1	2.3	
	posthemorrhgaic	201	26.4	13	6.5	
	postinfectious	26	3.4	1	3.8	
	posttraumatic	17	2.2	1	5.9	
	tumor	109	14.3	6	5.5	ns

P: chi square test, ns = not significant, IIH = idiopathic intracranial hypertension; NPH = normal pressure hydrocephalus

The second cohort (intervention group) comprised 499 procedures, of which 195 were in children. The M/F ratio of this group was 1.41 and 0.67 in children and in adults, respectively; the average age was 6.4 (± 5.5) and 49.4 (± 21.1) in children and in adults, respectively. The first shunt versus shunt revision ratio in children and adults was 0.51 and 1.05, respectively.

No complications attributable to the use of vancomycin were encountered in group 2.

### Infection rates overall

Overall, 33 shunt infections occurred in 762 procedures within 6 months after surgery (4,3%) (Table 2). The infection rate was 13.1% in children ≤1 year, 3.0% in children 1–17 years and 3.0% if ≥17 years. The difference of infection rates between ≤1 year and >1 year of age is significant (p = 0.001). Male patients had a higher shunt infection rate than females (5.6% versus 3.2%) but not significant (p = 0.11). Staff neurosurgeons had a higher infection rate than residents (4.8% vs. 3.3%, ns: p = 0.35). The etiology of hydrocephalus was studied for 10 different categories with infection rates of 0% to 6.5% but none was identified as a significant risk factor (Table 2). Although prematurity led to an infection rate of 16.2%, this was not significantly higher than in other children <1 year (11.3%, p = 0.545).

Shunt revision surgery came with a higher infection rate than new shunt insertion, but not significantly (5.2% vs. 3.3%, p = 0.214). However, when we look at shunt revisions we see that patients with surgery within a 7-day period before (8.9%) or within 30 days before (7.6%) the actual procedure, had higher infection rates than those with previous surgery > 1 month or >3 months before (resp. 4.0% and 2.8%). Previous surgery < 30 days shows a relatively strong association with an increased risk (7.6% versus 3.7%, p = 0.057).

### Infection rates in different study cohorts

In the control group, 18 shunt infections occurred (6.8%). In children, the shunt infection rate was 7.6% versus that of 6.1% in adults. Comparing patients aged <1 year versus >1 year, the shunt infection rate was 17.0% versus 4.6%. In the intervention group, there were a total of 15 shunt infections (3.0%). The shunt infection rate reduction of 6.8% to 3.0% was statistically significant (p = 0.023, absolute risk reduction 3.8%, relative risk reduction 56%). The shunt infection rate of children in the intervention group was 5.1% and in adults 1.6%. The shunt infection rate in patients aged <1 year to >1 year was 9.6% and 2.2%.

Although the overall reduction of shunt infection rate was significant, there was a remarkable change over time in the intervention group. During 2012 and 2013, the intervention group shunt infection rate was 5.6%, and 2014 and 2015 this was reduced to 1.1% (p = 0.006). Protocol compliance, however, was not a measured variable, while no protocol or institutional changes have been observed in this study period.

The shunt infection rate of new shunt implantations and shunt revisions in the control group was 5.2% and 8.2%, respectively; that in the intervention group was 2.3% (p = 0.197) and 3.6% (p = 0.063), respectively. Of the 30 patients with a shunt infection, 3 had a reinfection at new shunt insertion, which renders a re-infection rate of 10% after previous shunt infection. This is not significantly different from the overall cohort (p = 0.14). All 3 reinfections occurred in the control group, but the difference to the intervention group without reinfections was not significant (p = 0.22).

### Risk factors for shunt infection

Only factors that had a relative association (p<0.1) with shunt infection in univariate analysis (Table 3) were included for multivariate analysis. Prematurity, sex, previous shunt infection, type of surgeon and etiology were thus excluded as risk factors. Multivariate analysis demonstrated that only an age of the patient <1 year (OR 4.41; 95% CI 2.10–9.26) was a risk factor for infection and that our new protocol with vancomycin (OR 0.49; 95% CI 0.24–0.998) was a protective factor.

**Table 3:** Multivariate logistic regression analysis of risk factors for shunt infection.

Risk factors	No. of cases	Univariate	analysis	Multivariate	analysis
		OR	95% CI	OR	95% CI
Gender (female)	406	0.56	0.27–1.13		
Age <1 year	99	4.86	2.33–10.12	4.41	2.10–9.26
Prematurity (cases <1 year)	37	1.52	0.47–4.93		
New protocol	499	0.42	0.21–0.85	0.49	0.24–0.998
New shunt	338	0.62	0.29–1.29		
Surgeon (staff)	516	1.52	0.67–3.41		
Previous surgery (<1month)	132	2.16	1.00–4.66	1.84	0.83–4.07

### Time to infection

Overall, 23 shunt infections (70%) occurred early, within 30 days after surgery. In the control group (without topical vancomycin), 17 of 18 infections (94%) occurred within 1 month; in the intervention group, only 6 of 15 infections (40%) were early (p = 0.001). Median time to infection was 7 days (2–33) versus 38 (4–105) days. Cox proportional hazard regression analysis showed a hazard ratio (HR) of 0,47 (95% CI 0.24–0.93, p = 0.031) for the intervention group versus the control group, a HR of 4,38 (95% CI 2,17–8,84, p = 0,001) for age <1year and a HR of 1,86 (95% CI 0,88–3,96, p = 0,11) for previous surgery within 1 month.

### Microbiology

Table 4 lists the causative microorganisms found in the cultures, both for initial shunt placements and for revision surgery. No microorganisms were found in only two cases, although the clinical presentation and CSF clearly showed shunt infection. Thirteen infections (42% of known organisms) were caused by different species of coagulase-negative staphylococci (CoNS), and 7 times by *S*. *aureus* (23%). Although the numbers were low, they do not seem to show alterations in the spectrum of causative organisms after the introduction of vancomycin.

**Table 4:** Pathogen identified at shunt infection.

Control Group		Intervention Group	
Microorganism	No.	Microorganism	No.
Coagulase-negative staphylococci	7	Coagulase-negative staphylococci	6
*Staphylococcus aureus*	4	*Staphylococcus aureus*	3
*Streptococcus viridans*	1	*Streptococcus mitis*	1
*Enterobacter cloacae*	2	*Klebsiella oxytoca*	1
*Pseudomonas aeruginosa*	1	*Proprionibacterium acnes*	2
*Escherichia coli*	2	*Enterococcus faecalis*	1
No organism	1	No organism	1

The minimum inhibitory concentration (MIC) of vancomycin was determined for 10 out of 14 microorganisms in the intervention group. The MIC varied from 0,5–2 μg/ml and no vancomycin resistant strains were identified.

## Discussion

We studied the effect of adding topical vancomycin to a previously implemented shunt infection prevention protocol on the incidence of postoperative shunt infections. The shunt infection rate decreased significantly from 6.8% pre-implementation to 3.0% post-implementation and remained significant after correction for confounders in multivariate logistic regression analysis. The reduction was greater in adults than in children and decreased further during the study period to <2%; thus, shunt infections have become a marginal problem.

Although we did not monitor protocol violations, we discovered based on samples that the use of vancomycin was occasionally forgotten, especially in the first year. Efforts were made to increase protocol awareness and adherence in that time, especially in scrub nurses, who are the mainstay in executing the use of vancomycin. Repetitively emailing the neurosurgical staff and residents with reminders and the provision of motivational speeches to all contributing nursing staff subsequently made the new protocol a joint effort and joint responsibility, which may also have contributed to the improved results over the years. Without a formal compliance registration this remains, however, speculative.

Our results compare favorably with the results of other studies in which a strict prevention protocol was followed or in which AIC have been used [3–5, 13, 14, 16, 17]. Our study might be biased by better compliance to the shunt infection prevention protocol as a whole after the introduction of the protocol. However, the other components of this protocol had been introduced in 2004 and the shunt infection rate had been stable for many years before the study intervention, probably mirroring constant compliance. We noted that in the first half of our study, wound breakdown was a major factor in the development of shunt infection in infants. In the second half of the study, when our shunt infection rate dropped from 5.6% to 1.1%, we did not encounter a single wound breakdown or skin laceration. Paying particular attention to the mechanical factors of wound healing may thus contribute to a lower shunt infection rate. Obviously, this cannot be the explanation for the observed decrease in shunt infection rate among the adult patients. Only a single factor being changed in an existing protocol suggests that vancomycin was at least partially responsible for the observed effect.

Interestingly, the use of vancomycin shifted the occurrence of a shunt infection in time to a later period, with 60% of infections occurring after 1 month while only 6% of infections occurred after 1 month without vancomycin, and thus a prolonging of the time-to-infection due to the use of vancomycin. However, the spectrum of causative microorganisms did not change as a result of the use of vancomycin and there are no signs that remaining infections are the result of vancomycin resistant strains of bacteria.

### CSF shunt infection prevention

The mainstay of shunt infection prevention is surgery protocolization and preparation. The first and most impressive proof of the shunt infection rate-lowering effect of such a protocol was reported by Choux et al., who were able to reduce the shunt infection rate to 1% in a large cohort of pediatric patients [18]. Hardly anyone has reproduced this result. A main reason for this is that the strictness of their protocol conflicted with the typical logistics in teaching hospitals. The use this protocol would mean the absolute end of training for new residents, scrub nurses, anesthesiologists, as they would not be allowed to participate in the OR. Many elements of the protocol by Choux et al., however, have found their way into the myriad protocols currently used worldwide, and many hospitals have thus been able to lower their shunt infection rate.

There is, however, very little or even no hard evidence for each measure in these protocols separately, and it seems that it is the “package” that is effective [5, 11]. The motto “It is more important to do the same than to do it right” is relevant to many different medical processes, be it open heart surgery, treating sepsis in the intensive care unit, or transurethral resection of the prostate (TURP). Thus, decreasing treatment variation or practicing variation decreases error rates and costs and improves outcomes [19]. It is most likely this effect that reduces the shunt infection rate the most.

The North American Hydrocephalus Clinical Research Network (HCRN) further supports the importance of protocol compliance. It proved that an 11-step protocol significantly lowered the shunt infection rate and that protocol compliance was inversely related to shunt infection rate [5]. In a follow-up study on AIC, in which AIC were added as part of the standard protocol, the shunt infection rate remained the same [4]. It appears that the protocol as a package is of more importance than the use of AIC for reducing shunt infection rate.

Only by changing one step of a multistep protocol can one study the effect of a single measure. We have shown that adding a single step to an existing protocol (topical vancomycin) significantly reduced shunt infection rate.

### Intravenous and intrathecal antibiotics

A meta-analysis by Klimo et al. showed that the application of iv antibiotics has more or less become an internationally accepted standard with a moderate degree of clinical certainty that it lowers the shunt infection rate [10, 20], although the number of antibiotics used varies widely. There appears to be consensus that iv antibiotics should be given 15–30 minutes prior to skin incision, but whether antibiotics should be continued for one or more days after surgery is subject to wide practice variation. We use cefazolin, a first-generation cephalosporin, providing coverage for methicillin-susceptible *S*. *aureus* (>95% of *S*. *aureus* isolates in The Netherlands are methicillin-susceptible), and to a lesser extent, for Enterobacteriaceae.

Vancomycin is widely used intrathecally for treating shunt-related infections [21]. However, it is only sporadically used for preventing infection, both iv and intrathecally. Ragel et al. initiated the use of both gentamycin and vancomycin intrathecally for preventing infection and reduced the shunt infection rate from 5.4% to 0.4% [15]. Moussa and Mohamed used a similar antibiotic regimen in a small, prospective, randomized trial of children aged <1 year, but applied the antibiotics differently. Gentamycin and vancomycin were injected at the end of surgery into the ventricular reservoir and through the skin around the shunt hardware every 5 cm along the hardware, and in one group of patients, repeated after a week. They thus significantly reduced the shunt infection rate (from 30% to 2.5%) [22].

Although we did not copy these two protocols, the protocol of Ragel et al. was our incentive for altering our protocol and for introducing vancomycin intrathecally. However, protocol compliance is difficult to establish when many different surgeons have to adhere to a new protocol or when the protocol becomes too complicated, e.g., different dosages for children and adults. Therefore, we attempted to determine a means of applying vancomycin to air-exposed tissue/wounds and shunt hardware such that overdosing is always prevented even in the youngest babies. We therefore wanted to limit the vancomycin dosage to <10 mg in babies. This can be accomplished with a solution of 2 mg/ml vancomycin, as vancomycin is not introduced intrathecally, only inserted inside the shunt (volume of valve and tubing < 2 ml), while vancomycin solution–drenched gauzes come into contact with open wounds but hardly leave any solution behind after wound closure. After unpacking, the shunt tubing is immediately placed in the vancomycin solution and therefore only exposed to air for 2 seconds. All handling of the tubing is subsequently performed with instruments, wetted gloves, and wetted gauzes. In fact, our application of vancomycin is thus topical in the wound only.

There is no scientific basis for the manner in which we apply vancomycin. Practicality, safety, and simplicity were our reasons for limiting the use of vancomycin to this protocol. Our way of applicating vancomycin differs from intrathecal use of vancomycin in that intrathecal vancomycin only flushes the inside of the tube system, while topical vancomycin also flushes the outside of tubes and valves, thus those parts that are exposed to contamination.

### Antibiotic-impregnated catheters

Recent studies have investigated another form of topical prophylactic antibiotic application. In vitro studies have shown that impregnating catheters with antibiotics allow the prolonged release of antibiotics over weeks, which prevents bacterial colonization of these catheters. Prospective double-blinded multicenter randomized trials have established that AIC for external ventricular drainage (EVD) significantly reduced catheter infections [23, 24], although one such study did not report any difference [17]. Meanwhile, many different but relatively small studies showed decreased shunt infection rates with the use of AIC. Some of these studies achieved a statistically significant reduction of shunt infection rates, while others did not. Meta-analysis of these pooled data by different authors appear to prove that the effect of AIC is real and significant, although with only a class III level of evidence and level III recommendation [6, 9, 12, 14]. Others have shown that AIC might save lives, prevent infection-related neurological morbidity, and are also cost-effective [13, 25]. Therefore, AIC use has become increasingly popular, and some suggest that this should become standard of care.

However, there are some concerns as well. First, there may be the risk of bacterial resistance developing against the antibiotics used. Thus far, no evidence shows such a development [3], although the meta-analysis by Konstantelias et al. showed that antimicrobial shunt catheters were associated with higher risk for methicillin-resistant staphylococcus aureus (MRSA), nonstaphylococcal, and gram-negative bacterial infections, and thus a shift towards more virulent strains [6]. In 2 studies, however, shunt revision surgery of shunts in which in previous surgery used, an AIC led to an increased shunt infection rate of 11.7% [3]. The numbers are still too low to draw definitive conclusions, but follow-up studies in the coming years will shed light on this concern.

Another concern is the fact that the most impressive decrease of shunt infection rates is reported in centers with an offset of a relatively high shunt infection rates before changing to the use of AIC. In a British multi-center trial of 3 academic pediatric neurosurgical institutions, AIC significantly reduced the shunt infection rate overall, but there was no change in the shunt infection rate after AIC was introduced in one center with an already much lower shunt infection rate than the other 2 centers [16]. This suggests that there may be bias due to the use of shunt infection prevention protocol or just due to the increased attention placed on infection prevention. In most studies, the effect of AIC is compared to a retrospective series of shunt surgery, so that introducing AIC is part of a new infection prevention protocol or improved compliance with a previous protocol. A surgeon cannot be blinded to the yellow AIC when non-AIC are usually white, so there is bias. In at least one European neurosurgical center in which AIC significantly decreased that institute’s shunt infection rate, the shunt infection rate remained unchanged after discontinuation of the use of AIC (personal communication).

The HCRN has shown that adding AIC to an already proven effective 11-step protocol with strong protocol compliance did not further decrease the shunt infection rate [4].

In light of the above findings, it seems justified to conclude that it has not been established that AIC significantly decrease shunt infection rate and that a protocol effect on decreasing shunt infection rate is just as likely. More studies such as that performed by the HCRN, e.g., in Europe, are urgently needed. As the shunt infection rate-reducing effect of our protocol is of the same size or even better than that obtained with AIC, our protocol would also warrant the same international scrutiny through further studies. However, the incremental costs of vancomycin are $10 per surgery versus $400 for AIC, and thus our protocol could be an alternative to AIC and should be the first choice in developing and third-world countries. Using vancomycin instead of AIC saves our department more than €50,000 annually.

### Study limitations

Several limitations regarding our study should be considered. We retrospectively examined 2 consecutive patient cohorts, which may be subject to several types of bias. Changing a protocol in the second group may have led to protocol bias. However, the best results were obtained 2–3 years after changing the protocol and not immediately after, which was to be expected in the case of protocol bias. Also, the results were not monitored by all surgeons, but only by a single person (EJvL) who is one of the surgeons and thus not an independent investigator.

The primary outcome was shunt infection. Shunt infection leads to surgery or at least prolonged hospitalization. Outpatient treatment of a shunt infection does not go unnoticed by the neurosurgeon. Loss of patient follow-up as a result of treatment elsewhere does not occur in the landscape of Dutch neurosurgical care; therefore, shunt infections treated at a different hospital would not have contributed to observation bias. Missing or unregistered diagnoses of shunt infections are therefore unlikely to cause bias.

Although the new updated protocol was introduced uniformly to the neurosurgical staff and surgical nurses and was rather simple and strict, the compliance rate of protocol adherence is unknown. In a few cases, we found that vancomycin was forgotten during surgery. The reduction of shunt infection rate over time in group 2 suggests improved protocol compliance. In their HCRN studies, Kestle et al. showed that adherence to a protocol increases results and reduces shunt infection rate [5]. We cannot rule out that better adherence to the other aspects of the protocol instead of the addition of topical vancomycin may be (partially) responsible for the observed decrease in infection response rate. However, the increased time to infection in the intervention group offers a biological explanation for the protective effect of vancomycin rather than better adherence to the other aspects of the protocol.

## Conclusions

The use of 2 mg/ml intraoperative topical vancomycin (intrashunt and perishunt) in combination with a multistep shunt infection prevention protocol significantly reduces the shunt infection rate for internalized CSF shunts and this treatment may be a cheaper alternative to AIC use.
